# Comparison of [^68^Ga]Ga-DOTA-FAPI-04 and [^18^F]FDG PET/MRI in the Preoperative Diagnosis of Gastric Cancer

**DOI:** 10.1155/2023/6351330

**Published:** 2023-04-08

**Authors:** Tao Du, Shun Zhang, Xi-Mao Cui, Ren-Hao Hu, Hai-Yan Wang, Jian-Juan Jiang, Jun Zhao, Lan Zhong, Xiao-Hua Jiang

**Affiliations:** ^1^Department of Gastrointestinal Surgery, Shanghai East Hospital, School of Medicine, Tongji University, Shanghai 200120, China; ^2^Department of Nuclear Medicine, Shanghai East Hospital, School of Medicine, Tongji University, Shanghai 200120, China; ^3^Department of Digestive Medicine, Shanghai East Hospital, School of Medicine, Tongji University, Shanghai 200120, China

## Abstract

**Purpose:**

Our objective was to compare the value of positron emission tomography/magnetic resonance imaging (PET/MRI) with the new imaging agent [^68^Ga]Ga-DOTA-FAPI-04 and the traditional imaging agent [^18^F]FDG for the preoperative diagnosis of gastric cancer.

**Methods:**

Forty patients with gastric cancer diagnosed by gastroscopy in gastrointestinal surgery at our hospital from June 2020 to January 2021 were analyzed. All patients underwent simultaneous [^68^Ga]Ga-DOTA-FAPI-04 and [^18^F]FDG PET/MRI. The standard uptake value (SUV), fat removal standard uptake value (SUL), and diagnostic sensitivity, specificity, and accuracy for primary and metastatic lesions were compared, and their diagnostic value for different lymph node dissection stages was analyzed.

**Results:**

The median age of the patients in this cohort was 68 years. Twenty-nine patients underwent surgery, and 11 patients underwent gastroscopic biopsy. The SUV_max_ of primary lesions in the FDG group and the FAPI group was 5.74 ± 5.09 and 8.06 ± 4.88, respectively (*P* < 0.01); SUL_max_ values were 3.52 ± 2.80 and 5.64 ± 3.25, respectively (*P* < 0.01). The SUV_max_ of metastases in the two groups was 3.81 ± 3.08 and 5.17 ± 2.80, respectively (*P* < 0.05). The diagnostic sensitivities for primary lesions in the FDG group and the FAPI group were 0.72 and 0.94, respectively (*P* < 0.05). Combined with postoperative pathological staging, there was no difference in diagnostic sensitivity and specificity of lymph node staging between the FDG and FAPI groups (*P* > 0.05).

**Conclusion:**

Compared with the traditional imaging agent, [^68^Ga]Ga-DOTA-FAPI-04 has better diagnostic efficiency but no substantial advantage for preoperative lymph node staging.

## 1. Introduction

Cancer-associated fibroblasts (CAFs) are the main components of the matrix around epithelial cancer cells and can selectively produce fibroblast activation protein (FAP). FAP is highly expressed in a variety of epithelial cancers, such as gastric cancer, colorectal cancer, esophageal cancer, and ovarian cancer, but it is almost not expressed in the matrix of normal tissues [1, 2]. Based on this characteristic, fibroblast activation protein inhibitor (FAPI) has been used as the imaging agent for positron emission tomography (PET) in the last few years, for which [^68^Ga]Ga-FAPI-04 was developed [3]. Because stromal cells account for 90% of the total weight of tumors, cell matrix-based targeted PET may be more sensitive than glucose metabolism PET imaging. Studies have shown that [^68^Ga]Ga-FAPI-04 has stable performance and can reflect some characteristics of different solid tumors [4, 5]. Other studies have shown that [^68^Ga]Ga-FPAI-04 positron emission tomography/computed tomography (PET/CT) results in clearer contours and a higher target-to-background ratio than [^18^F]FDG PET/CT for solid tumors [6]. In addition, [^68^Ga]Ga-FAPI-04 PET/CT has a higher uptake value and diagnostic accuracy for some suspicious tumors that cannot be characterized by [^18^F]FDG PET/CT [7].

Gastric cancer is a common disease, and its occurrence is related to many factors, such as HP and garlic [8, 9]. PET/MRI has been widely used in the evaluation of gastric cancer in the last few years. It has the advantages of better soft tissue contrast, functional imaging, and less ionizing radiation. Its disadvantage is also obvious: it is easily affected by respiration and gastric peristalsis during imaging, which results in artifacts. It was found that [^18^F]FDG PET/MRI has better advantages in preoperative TNM staging than PET/CT [10]. Another study showed that multidetector CT (MDCT) combined with [^18^F]FDG PET/MRI improves the diagnostic accuracy of a preoperative M-stage in recurrent gastric cancer and has advantages in evaluating the resectability of lesions [11]. At present, research on [^68^Ga]Ga-FAPI-04 PET/MRI in the diagnosis of gastric cancer is very limited. This study sought to compare the value of [^68^Ga]Ga-FAPI-04 and [^18^F]FDG PET/MRI for the diagnosis of gastric cancer.

## 2. Materials and Methods

### 2.1. Patients

This study is a prospective cohort study that included 40 patients with gastric cancer diagnosed by gastroscopy in the Department of Gastrointestinal and Anorectal Surgery of our hospital from June 2020 to January 2021. The inclusion criteria were as follows: diagnosed with gastric cancer by gastroscopy, age between 18 and 80 years, and no contraindication for PET or MRI. The exclusion criteria were as follows: combined with other tumors, accompanied by pyloric obstruction or bleeding or other severe organ dysfunction. All patients underwent simultaneous [^68^Ga]Ga-FAPI-04 and [^18^F]FDG PET/MRI.

### 2.2. PET/MRI Imaging

All patients underwent [^68^Ga]Ga-FAPI-04 (d1) and [^18^F]FDG PET/MRI (d3) in turn, with an interval of more than 48 hours. All examinations were conducted in the nuclear medicine discipline of East Hospital affiliated with Tongji University according to the standard process. [^18^F]FDG PET/MRI : the patient fasted for 12 h before the examination, with blood glucose < 11 mmol/L. After lying flat for 20 minutes, [^18^F]FDG (5.5 MBq/kg) was intravenously injected, and the patient drank 1000 ml of water after resting for 40 minutes. The scanning range was whole-body from the head to the groin for approximately 30 minutes. [^68^Ga]Ga-DOTA-FAPI-04 PET/MRI: [^68^Ga]Ga-DOTA-FAPI-04 was injected at the same concentration, and the remaining steps were the same as those for [^18^F]FDG PET/MRI.

### 2.3. Imaging Review

PET/MRI images were analyzed by two nuclear medicine physicians with experience in PET/MRI for more than 2 years. The standard uptake value (SUV), fat removal standard uptake value (SUL), diagnostic sensitivity, specificity, and accuracy of primary and metastatic lesions were measured, and their diagnostic value for different lymph node dissection stages was analyzed. The participants were not given information about the other PET/MRI scan. In case of disagreement in diagnosis, the two doctors discussed and reached a consensus.

The main function of PET is to detect a lesion, and MRI images are used to confirm whether the hypermetabolic area is the tumor. TNM staging of gastric cancer was determined by referring to the 8th edition of the AJCC gastric cancer staging system. The criteria for lymph node metastasis of gastric cancer were as follows: shortest diameter > 5 mm, necrotic signs in the center, high DWI signal and low ADC signal, and higher metabolism than the background.

### 2.4. Statistical Analysis

Data are expressed as the mean ± standard deviation, and statistical analyses were conducted using SPSS 22.0 software. *T* tests were used to compare measurement data between two groups, and the chi-square test was used to compare count data. Sensitivity, specificity, and accuracy were compared by the McNemar test. *P* < 0.05 was considered statistically significant.

## 3. Results

### 3.1. Patients' Clinical Characteristics

A total of 40 patients with a median age of 68 years were enrolled in this study, including 32 males and 8 females, with an average BMI of 22.1 ± 2.61. A total of 29 patients underwent radical resection, and 11 underwent biopsy only. Thirty-six cases of gastric cancer and 4 cases of benign diseases were confirmed by pathology. Thirteen patients had CEA > 5 ng/ml, and 10 patients had CA199 > 37 ng/ml. There were 18 cases with HER2 expression (+∼+++), 5 cases with dMMR, and 2 cases with PDL-1 percentage > 5% (Table 1).

### 3.2. Uptake of [^18^F]FDG and [^68^Ga]Ga-DOTA-FAPI-04

**Figure 1:**
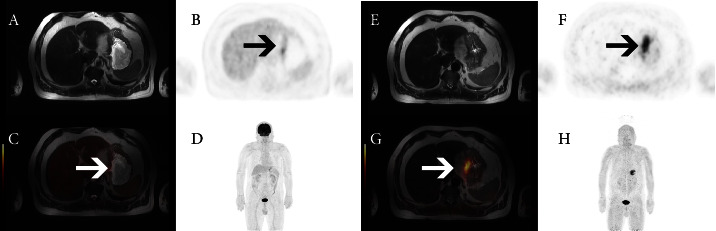
A 64-year-old male patient with gastric cancer diagnosed as gastric fundus tumor by gastroscopy. FDG-PET MRI showed a slight increase in FDG metabolism, with an SUVmax of 3.74 and no increase in metabolism in perigastric lymph nodes (A-D). FAPI-PET/MRI showed obvious thickening of the cardia, gastric fundus and lesser curvature of the gastric body, and an abnormal increase in FAPI uptake (arrow), with an SUV_max_ of 11.2 (E-H). Small lymph node shadows were detected behind the lesser curvature of the stomach and the pancreas, and there was no FAPI uptake.

**Figure 2:**
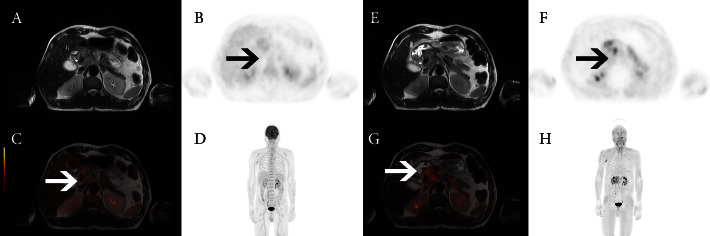
A 63-year-old male patient with gastric cancer, a gastroscopy showed that gastric cancer had invaded the lower esophagus, with perigastric infiltration. PET/MRI showed abnormal increases (arrow) in FDG metabolism (A-D) and FAPI uptake (E-H).

All patients underwent [^18^F]FDG and [^68^Ga]Ga-DOTA-FAPI-04 PET/MRI (Figures 1 and 2). The results showed that the maximum SUVs (SUV_max_) of primary lesions in the [^18^F]FDG group and [^68^Ga]Ga-DOTA-FAPI-04 group were 5.74 ± 5.09 and 8.06 ± 4.88, respectively (Table 2, *P* < 0.01); maximum SUL (SUL_max_) values were 3.52 ± 2.80 and 5.64 ± 3.25, respectively (*P* < 0.01). For metastatic lesions, SUV_max_ values were 3.81 ± 3.08 and 5.17 ± 2.80 in the [^18^F]FDG group and [^68^Ga]Ga-DOTA-FAPI-04 group, respectively (*P* < 0.05); SUL_max_ values were 2.65 ± 2.21 and 3.80 ± 1.74, respectively (*P* > 0.05). There was no substantial difference between SUV_max_ or SUL_max_ between the two groups (*P* > 0.05).

### 3.3. Diagnostic Efficiency of Primary and Metastatic Lesions

The sensitivity, specificity, and accuracy of [^18^F]FDG for the diagnosis of primary lesions were 0.72, 0.25, and 0.78, respectively; those for [^68^Ga]Ga-DOTA-FAPI-04 were 0.94, 0, and 0.85, respectively. There was a substantial difference in sensitivity (*P* < 0.05) but no difference in specificity and accuracy (*P* > 0.05). The sensitivity, specificity, and accuracy of [^18^F]FDG for the diagnosis of metastatic lesions were 0.33, 0.82, and 0.62, respectively; those for [^68^Ga]Ga-DOTA-FAPI-04 were 0.58, 0.71, and 0.66, respectively, with no substantial difference (Table 3, *P* > 0.05).

### 3.4. Diagnostic Efficiency of Lymph Node Staging

The lymph node staging of all 40 patients in the two groups is shown in Tables 4 and 5. Based on the postoperative pathology of 29 patients who underwent surgical resection, the numbers of N0, N1, N2, and N3 cases were 17, 6, 1, and 5, respectively. Correspondingly, the numbers of N0, N1, N2, and N3 were 21, 3, 4, and 1 diagnosed in the [^18^F]FDG group and 18, 3, 5, and 3 in the [^68^Ga]Ga-DOTA-FAPI-04 group, respectively (Table 4). A total of 7 patients received preoperative neoadjuvant chemotherapy.

The sensitivity, specificity, and accuracy of [^18^F]FDG were 0.33, 0.82, and 0.62, and that of [^68^Ga]Ga-DOTA-FAPI-04 were 0.58, 0.71, and 0.66, respectively. The sensitivity, specificity, and accuracy of the two groups in the N1 phase were 0 and 0.33, 0.77 and 0.81, and 0.69 and 0.76, respectively, those of the N2 phase were 0 and 0, 0.96 and 0.96, and 0.83 and 0.79, and those of the N3 stage were 0 and 0, 0.82 and 0.81, and 0.79 and 0.72. However, there were no substantial differences between the two groups (Table 5, *P* > 0.05).

## 4. Discussion

The standard treatment of gastric cancer depends on accurate preoperative staging [12], which is also important for metastatic gastric cancer. The purpose of this prospective study was to compare the diagnostic value of the new imaging agent [^68^Ga]Ga-DOTA-FAPI-04 and the traditional agent [^18^F]FDG. Our results showed that the maximum uptake value of [^68^Ga]Ga-DOTA-FAPI-04 was better than that of [^18^F]FDG, with sensitivity in the diagnosis of primary lesions of gastric cancer being better than that of [^18^F]FDG. Nonetheless, there was no substantial difference in the sensitivity and specificity of lymph node staging between the two groups.

The uptake values of SUV_max_ and SUL_max_ in the FAPI group were higher than those in the FDG group, which was consistent with the results of Chen et al. [13]. Some studies have shown that uptake of [^18^F]FDG is lower in diffuse gastric cancer, gastric mucinous adenocarcinoma, and signet ring cell carcinoma, which affects the diagnosis of gastric cancer [14]. Therefore, our results suggest that [^68^Ga]Ga-DOTA-FAPI-04 may be able to compensate for this deficiency, even though we did not conduct a subgroup analysis of gastric cancer histology type in this study. Further diagnostic analysis found that the sensitivity of [^68^Ga]Ga-DOTA-FAPI-04 for primary lesions was 0.94, which was considerably higher than that of [^18^F]FDG. This was consistent with the study of Guo et al. [15]. We speculate that this is related to the high uptake rate of [^68^Ga]Ga-DOTA-FAPI-04. Because all patients were diagnosed with gastric cancer by gastroscopy, specificity could not be compared for the two groups. In the comparison of diagnostic efficacy for metastases, we found that the sensitivity of the FAPI group tended to increase (0.58 and 0.33, respectively), but there was no substantial difference in the *P* value. We speculate that this may have been due to the inconsistent judgment criteria for positive lymph nodes. In previous studies, the cutoff value of a lymph node's short diameter (usually 5 mm) was used as the criterion for determining positive lymph nodes [16, 17]. However, inflammation can also lead to lymph node enlargement, and even metastatic lymph nodes do not necessarily show a volume increase. These factors may affect the diagnostic efficacy of PET/CT or PET/MRI for lymph node metastasis. In addition, recent studies have shown that lymph node metastasis can be identified by DWI sequences and ADC images [18]. Therefore, the MRI signal combined with SUV_max_ was used to identify lymph node metastasis in this study. Although our data show that SUV_max_ of the FAPI group and FDG group was not significantly different, we speculate that this may have been due to the small number of cases or selection bias. However, the increasing trend of SUV_max_ in the FAPI group suggests that [^68^Ga]Ga-DOTA-FAPI-04 had certain advantages in determining lymph node metastasis in gastric cancer.

We attempted to compare the diagnostic efficiency of the two imaging agents for different N stages of gastric cancer, though the results showed that the difference in diagnostic sensitivity and specificity between the two groups was not significant at N0 or N1-N3. We speculate the following reasons in addition to the factors of positive diagnostic criteria for lymph nodes. First, inflammation in lymph nodes may lead to increased uptake. Indeed, it has been reported that inflammation may lead to an abnormal increase in FDG metabolism in lymph nodes, thus increasing the false-negative rate [19]. Second, there may have been selection bias with regard to the patients enrolled. Only 41.4% (12/29) of the patients had lymph node metastasis confirmed by postoperative pathology. The small number of N1, N2, and N3 stage cases precluded analysis. In addition, only 29 of 40 patients received surgical treatment, and the limited number of cases affected N-stage diagnostic efficiency. In addition, we believe that the use of FAPI is beneficial to improve the preoperative N stage of patients, as well as perioperative management including neoadjuvant chemotherapy and postoperative follow-up. The results of this study show that the uptake value of [^68^Ga]Ga-DOTA-FAPI-04 in gastric cancer is higher than that of [^18^F]FDG; its diagnostic efficiency for primary lesions is also higher, which indicates that [^68^Ga]Ga-DOTA-FAPI-04 MRI may be a more effective method for the diagnosis of gastric cancer. Of course, this study also had some limitations, such as the small number of enrolled cases and the lack of data on tumor T staging. In addition, the number of patients with dMMR and PDL-1 positivity was relatively small, and there was a lack of subgroup analysis. Prospective studies with a larger number of patients in the future may provide more evidence.

## Figures and Tables

**Table 1:** Patient characteristics.

Total patients	40
Median age	68
BMI	22.1 ± 2.61
Sex	
Male	32
Female	8
Treatment	
Biopsy only	11
Resection	29
Histology	
Adenocarcinoma	23
Signet ring cell carcinoma	13
Benign	4
CEA > 5 ng/ml	13
CA199 > 37 ng/ml	10
Tumor diameter	3.6 ± 1.56
Her2	
++/+++	10
+	8
−	8
None	14
MMR	
dMMR	5
pMMR	35
PDL-1	
>= 5%	2
< 5%	38

**Table 2:** Comparison of [^18^F]FDG and [^68^Ga]Ga-DOTA-FAPI-04 uptake in gastric cancer.

	Primary lesions		Metastatic lesions	
	SUV_max_	SUL_max_	SUV_max_	SUL_max_
FDG	5.74 ± 5.09	3.52 ± 2.80	3.81 ± 3.08	2.65 ± 2.21
FAPI	8.06 ± 4.88	5.64 ± 3.25	5.17 ± 2.80	3.80 ± 1.74
*P* value	0.004	0.005	0.018	0.08

**Table 3:** Diagnostic performances of [^68^Ga]Ga-DOTA-FAPI-04 and [^18^F]FDG PET/MRI in assessment of gastric tumor and lymph node involvement.

Basis of analysis and modality	Imaging diagnosis	Pathologic diagnosis		Sensitivity	Specificity	Accuracy
Primary lesions		+	−			
FDG	+	26	3	0.72	0.25	0.68
	−	10	1			
FAPI	+	34	3	0.94	0.25	0.88
	−	2	1			
*P* value				0.02	1.00	0.07

Metastatic lesions						
FDG	+	4	3	0.33	0.82	0.62
	−	8	14			
FAPI	+	7	5	0.58	0.71	0.66
	−	5	12			
*P* value				0.25	0.50	0.79

**Table 4:** Clinical stage and pathological stage of patient.

N stage	Standard	N		Neoadjuvant chemotherapy
		FDG	FAPI	
N0	17	21	18	0
N1	6	3	3	2
N2	1	4	5	2
N3	5	1	3	3

**Table 5:** Diagnostic results of N stage between [^68^Ga]Ga-DOTA-FAPI-04 and [^18^F]FDG PET/MRI according to the gold standard.

		Sensitivity (%)	Specificity (%)	Accuracy (%)
N0	FDG	0.82	0.63	0.62
	FAPI	0.71	0.58	0.66
	P	1.00	0.50	0.56

N1	FDG	0.00	0.77	0.69
	FAPI	0.33	0.81	0.76
	P	None	1.00	0.27

N2	FDG	0.00	0.96	0.83
	FAPI	0.00	0.96	0.79
	P	None	1.00	0.47

N3	FDG	0.00	0.82	0.79
	FAPI	0.00	0.81	0.72
	P	None	0.50	0.25

## Data Availability

The data used to support the findings of this study are available from the corresponding author upon request.
